# Results Reporting and Registry-Linked Publications Among Extracorporeal Membrane Oxygenation Studies Registered on ClinicalTrials.gov: A Cross-Sectional Meta-Research Study

**DOI:** 10.7759/cureus.117548

**Published:** 2026-10-06

**Authors:** Oscar Mingtian Li, Yijiang Li

**Affiliations:** 1 Cardiac/Thoracic/Vascular Surgery, Zweistein Innovation Lab, Active Care and Education in Sports and Outreach Foundation (ACESO Foundation), Dublin, USA; 2 Cardiology, Zweistein Innovation Lab, Active Care and Education in Sports and Outreach Foundation (ACESO Foundation), Dublin, USA

**Keywords:** clinical trial registry, clinicaltrials.gov, critical care, ecmo, extracorporeal life support, extracorporeal membrane oxygenation, meta-research, publication bias, results reporting

## Abstract

Extracorporeal membrane oxygenation (ECMO) research has expanded rapidly, but how often completed ECMO studies make their results publicly available is not known. This cross-sectional meta-research study examined ECMO study records on ClinicalTrials.gov at a census date of 13 September 2026. A field-scoped query of the registry's application programming interface returned 524 records, of which 493 met prespecified eligibility criteria after duplicate model-assisted screening with every decision checked by the first author. The primary outcome was the proportion of completed studies with a primary completion date at least 12 months before the census date that had summary results posted on the registry. Registry-linked PubMed references were classified by article type. Of the 493 studies, 268 (54.4%) were observational and 471 (95.5%) had a non-industry lead sponsor. In the 184-study 12-month-observable completed cohort, 14 had posted summary results (7.6%, 95% CI 4.6 to 12.4), and one had done so within 12 months of primary completion. Posting was more frequent in interventional than observational studies (17.6% versus 1.7%, risk difference 15.9 percentage points, 95% CI 7.5 to 26.7). Sixty-one studies (33.2%) carried at least one registry-linked PubMed reference, and 60 (32.6%) were linked to an article reporting their results. All four records meeting a post hoc registry-based approximation of applicable clinical trial status had posted results, but this subgroup was too small, and its classification too uncertain, to support any inference about regulation. Summary-results posting was uncommon among completed ECMO studies, a majority of which are observational and therefore outside the scope of United States statutory reporting requirements.

## Introduction and background

Extracorporeal membrane oxygenation (ECMO) has become an established component of critical care, with international registry activity describing more than one hundred thousand supported patients and randomised trials in respiratory failure, refractory cardiac arrest and cardiogenic shock [1-8]. Interpreting that evidence base depends on a condition rarely examined: that the studies conducted in the field reach the public record.

Three distinct steps are involved, and this study keeps them separate. Registration makes a study's existence and design public before it reports. Posting summary results in the registry's structured results database makes its findings public in a standard format, independent of journal publication [9]. Publication in a peer-reviewed journal is a third, separate route. In the United States, submission of summary results became a legal requirement for a defined class of applicable clinical trials under the Food and Drug Administration Amendments Act (FDAAA) Final Rule, effective 18 January 2017 [10,11]. That rule is not the only source of an expectation to report: funders, institutions and journals set their own policies, and the absence of an FDAAA duty does not mean the absence of any obligation.

Audits in other fields have found results reporting on ClinicalTrials.gov incomplete among trials subject to the statute [12-16], and posted results often appear later than, and sometimes differ from, the corresponding journal publication [17,18]. Recent registry analyses in neighbouring fields report the same pattern: underreporting of results in more than 90% of interventional sepsis trials [19], only 16.5% of stroke trials reporting within 12 months [20], frequent discontinuation and non-publication of heart failure trials [21], and substantial waste among terminated coronavirus disease 2019 (COVID-19) trials [22]. Registry analyses of out-of-hospital cardiac arrest [23] and cardiogenic shock [24] trials have described their design and publication status, but none has examined results reporting across ECMO research as a whole. The cumulative consequence of inaccessible research has been argued to constitute avoidable waste [25].

Whether these findings extend to ECMO is not known. The field may differ in composition: we hypothesised that ECMO research registered on ClinicalTrials.gov would be predominantly investigator-initiated and observational, and that much of it would therefore fall outside the class of applicable clinical trials to which the Final Rule attaches. The present analysis tests that composition directly rather than assuming it.

Because only registered records were examined, this study cannot estimate what proportion of ECMO research is registered. Its primary objective was to estimate the proportion of completed ECMO studies registered on ClinicalTrials.gov that had posted summary results at least 12 months after primary completion. Secondary objectives were to describe the characteristics of registered ECMO studies; the time from primary completion to results submission and posting; variation in posting across recorded study characteristics; the frequency of, and stated reasons for, discontinuation; and the presence and type of registry-linked PubMed references. An exploratory post hoc objective was to describe posting among the few records approximating applicable clinical trial status.

## Review

Materials and methods

Design, Protocol and Census Date

This was a cross-sectional meta-research study of records held on ClinicalTrials.gov. The unit of analysis was the registered study record, identified by its National Clinical Trial (NCT) number, and every field was read as it stood on a single census date, 13 September 2026. Reporting follows the Strengthening the Reporting of Observational Studies in Epidemiology (STROBE) statement for cross-sectional studies [26], with items of the REporting of studies Conducted using Observational Routinely-collected Data (RECORD) statement consulted because the data are routinely collected registry records [27]. No clinical findings were extracted or synthesised, so the Preferred Reporting Items for Systematic Reviews and Meta-Analyses (PRISMA) statement and a study-level risk-of-bias assessment do not apply; the risks of measurement error and misclassification specific to a registry audit are addressed under limitations.

The protocol, covering the search, eligibility criteria, variable definitions, outcomes, analyses and figures, was written before any record was retrieved and was not registered. Its file edit history was not preserved, and the file carries a deviation log appended after retrieval, so its prospective specification cannot be independently verified; it is presented as a documented but unregistered protocol. The protocol, the dated deviation log, and all study files are deposited in a public repository [28]. The protocol prespecified the variable categories, the precedence orders for condition and discontinuation coding, and the rule for computing enrolment tertiles; the pooling of small regions for comparative analysis and the post hoc subgroup described below were decided after the data were observed and are recorded as deviations. The study used only public registry records and involved no human participants and no identifiable individual data, so ethics review was not required.

Data Source and Search

Records were retrieved on 13 September 2026 at 07:20 UTC from the ClinicalTrials.gov application programming interface, version 2.0.5 [29], endpoint https://clinicaltrials.gov/api/v2/studies, using format=json, countTotal=true and pageSize=1000, following nextPageToken until absent. The screened corpus was defined by the parameter filter.advanced applying the term set ("extracorporeal membrane oxygenation" OR ECMO OR "extracorporeal life support" OR ECLS OR "extracorporeal cardiopulmonary resuscitation" OR ECPR) to six fields: AREA[BriefTitle], AREA[OfficialTitle], AREA[InterventionName], AREA[InterventionOtherName], AREA[ConditionSearch] and AREA[ArmGroupLabel]. Matching is performed by the registry server and is case-insensitive; the ECLS abbreviation was retained, and records retrieved through an unrelated sense of an abbreviation were excluded at screening. The query returned 524 records. The complete unmodified response was saved, with its retrieval timestamp and a SHA-256 checksum, as the immutable input to every analysis; the exact query strings and retrieval code are deposited [28].

A broader all-field query on four of the six terms returned 1,499 records. It was run to gauge how many records mention ECMO only in passing and was not the screened corpus. Because a field-scoped search can miss eligible studies that mention ECMO only in summary or description text, a random sample of 100 of the 979 records returned by the broad query but not by the field-scoped query (numpy default_rng, seed 20260913) was screened against the same eligibility criteria after peer review.

Eligibility Criteria and Screening

A record was included if ECMO, ECLS or ECPR was the intervention under evaluation or a component of it (code I-i); the defining feature of the enrolled population, meaning patients receiving, weaning from, or formally evaluated for such support (I-ii); or the object of study in its own right, including the circuit, cannula, oxygenator, anticoagulation, transport, and staffing, training or organisation of care (I-iii). A record was excluded under the first applicable of six codes: ECMO only in the eligibility criteria (E1); ECMO only as one of several outcome events, as background, or as a registry keyword (E2); expanded access (E3); duplicate registration (E4); no human participants (E5); or withdrawal with no classifiable content (E6). Three rules fixed during screening and applied to all records are recorded as a deviation: a record was included under I-ii only if an arm or cohort was explicitly defined by ECMO receipt; ECMO appearing as an outcome event was E2 unless an inclusion code independently applied; and naming ECMO in the title as part of what was studied established inclusion.

Screening was performed in two independent passes, each assisted by a large language model (Claude, Anthropic, California, US). The first pass flagged a candidate code with a verbatim supporting quotation for every record; every quotation was verified programmatically as a character-exact substring of the field it was attributed to. The second pass was a separate run over all 524 records from the saved response, blind to the first pass; it followed the first rather than running in parallel, and duplicate screening is recorded as a deviation because the original protocol specified a single reviewer assisted by a model pre-pass. No calibration exercise or pilot screen was performed. The two passes agreed on 514 of 524 records (raw agreement 0.981; Cohen's kappa 0.823, 95% CI 0.715 to 0.932). The first author (OML) then checked every one of the 524 decisions against the record text and adjudicated the 10 disagreements, and the decisions analysed are those human-checked decisions. The model output, both passes, the adjudication reasons and the verbatim quotations are deposited [28].

Variables and Operational Definitions

Every variable was taken from a named field of the registry response. Results posting was defined by the top-level Results field. The date of results posting was protocolSection.statusModule.resultsFirstPostDateStruct, and the date of first submission was statusModule.resultsFirstSubmitDate; the two are distinguished because a legal deadline, where one applies, concerns submission, whereas public posting follows the registry's quality-control review. Delayed-submission certifications were identified from statusModule.dispFirstSubmitDate. Primary completion date, overall status and the why-stopped text came from the status module; study type, phase, allocation and enrolment from the design module; intervention types from the arms and interventions module; lead sponsor name and class from the sponsor module; locations from the contacts and locations module; and derived PubMed references from referencesModule entries of type DERIVED. The complete field dictionary is available online [28].

Lead sponsor class is the registry's own agency class, collapsed to industry, National Institutes of Health, other government, and other, the last absorbing the network and individual classes. Because the registry's other class is not validated as academic, lead-sponsor names in that class were classified after peer review as university, hospital or research institute; other institution or network; named individual investigator; or commercial company. A non-industry lead sponsor does not exclude industry funding or collaboration. Region was assigned from listed location countries: records listing locations in more than one country were classified as multinational and records listing none as not reported. For comparisons, regions other than Europe, East Asia and North America were pooled with multinational and not-reported records so that no stratum fell below eight studies. Condition category and discontinuation reason were each assigned by a single prespecified precedence order, and enrolment was divided into tertiles at cut points computed from the included cohort (36 and 100 participants). Partial dates were imputed to the fifteenth of the month when the day was missing and to 1 July when only the year was given.

Outcomes

The primary outcome was the proportion of studies with posted summary results in the 12-month-observable completed cohort: included studies with status completed and a primary completion date on or before 13 September 2025. The outcome measured public availability on the registry at the census date. It is not a measure of legal compliance: most records are not applicable clinical trials, and delayed-submission certifications and extensions, where they exist, could not be evaluated from registry fields beyond the certification date field described above.

Secondary outcomes were the interval from primary completion to results first submitted and first posted; posting across study type, randomisation, region, involvement of children and enrolment tertile; discontinuation, meaning terminated, withdrawn or suspended status, with the why-stopped text coded into nine prespecified categories; and the presence of at least one derived PubMed reference. After peer review, every derived PubMed reference in the cohort was classified from its PubMed record as a primary results report of the registered study, a secondary, post hoc or follow-up analysis, a protocol or design paper, or not a report of the registered study. Derived references are indexed links, not a comprehensive search of the literature, and no bibliographic search by National Clinical Trial (NCT) number, title or investigator was performed; the publication outcome is therefore a registry-linkage indicator and not a publication rate.

Post hoc Subgroup Approximating Applicable Clinical Trial Status

A subgroup was defined after the primary analysis and is exploratory. A record entered it only if it was interventional, had a primary completion date on or after 18 January 2017, listed at least one United States location, and had either a drug or biologic intervention with a phase list including phase 2, 3 or 4, or a device intervention. Applicable clinical trial status turns on facts the registry does not record, including Food and Drug Administration jurisdiction over the product, investigational new drug or device exemption status, device feasibility status and the responsible party's own determination, and the transition provisions of the rule are more complex than a single date. The subgroup is therefore an approximation that can both admit records that are not applicable clinical trials and omit records that are.

Statistical Analysis

Proportions were reported with Wilson score 95% confidence intervals [30]. For each comparison family, risk differences against a stated reference level were reported with Newcombe hybrid score 95% intervals [31], and one hypothesis test was reported per family: Pearson's chi-square test where expected counts allowed, Fisher's exact test for two-by-two tables, and a Monte Carlo permutation approximation to the exact test (50,000 replicates, seed 20260913) for larger tables. For the exact tests, the Pearson chi-square statistic was reported only as a description of the contingency table. All comparisons were exploratory, unadjusted for multiplicity, and confounded by one another.

The prespecified multivariable logistic regression, with sponsor class, study type, log enrolment, involvement of children, region and start year as covariates and a prespecified drop order to apply if events per variable fell below ten, was not computable because of complete separation. After peer review, Firth's penalised logistic regression [32] was added as a sensitivity analysis and not as a replacement for the prespecified model. Two Firth models were fitted. The first used the most reduced prespecified covariate set, which was lead sponsor class and log enrolment. The second used study type, North American location and log enrolment. For both models, 95% confidence intervals were calculated by the profile penalised-likelihood method.

Time from primary completion to results first posted was estimated with the Kaplan-Meier product-limit estimator, implemented directly from the product-limit formula in NumPy. The time origin was the recorded primary completion date, with partial dates imputed as above; the event was the results first posted date; records without posted results were censored at the census date. Because no competing event was modelled, the curve was reported as the Kaplan-Meier estimated proportion posting.

Sensitivity analyses recomputed the primary outcome using the earliest and latest calendar dates compatible with each partial primary completion date, excluding partially dated records, reversing all 10 screening adjudications, and widening the denominator to include terminated studies and studies of unknown status whose primary completion fell within the window.

The original analysis used Python 3.13.15 with pandas 2.3.3, NumPy 2.5.3, SciPy 1.18.1, statsmodels 0.15.0 and Matplotlib 3.11.1. After peer review, the complete pipeline was re-executed from the saved registry response in a separate environment (Python 3.11.15, pandas 3.0.2, NumPy 2.4.4, SciPy 1.17.1, statsmodels 0.15.0, Matplotlib 3.10.9), reproducing every previously reported value, and the revision analyses and revised figures were produced there. Pandas, NumPy, SciPy and Matplotlib are NumFOCUS-sponsored projects (Austin, Texas, United States); statsmodels is an independent open-source project. All figures were drawn programmatically in Matplotlib, and no figure was generated by an artificial intelligence image tool. The analysis code, environment specification and every computed statistic in machine-readable form are available online [28].

Artificial Intelligence Use

Claude (Anthropic, PBC, California, US) was used through the Claude Science application on 12 and 13 September 2026 and through the Claude application on 26 and 27 September 2026, for four purposes: assembling candidate references, each verified against PubMed or Crossref; the two model-assisted screening passes; a first-pass classification of the linked PubMed references, the broad-search sample and a second coding of the discontinuation reasons after peer review; and authoring analysis code and drafting text. Public registry records and the authors' protocol were supplied to the tool; no personal or identifiable data were shared. The exact screening instruction and the model build identifier were not retained, which limited reproduction of the model step but not of the analysis, because every screening decision analysed was checked by the first author and every model decision is deposited. The three post-review classifications (the linked PubMed references, the broad-search sample and the second coding of discontinuation reasons) had not been checked record by record by a human reviewer at submission. Quality control of the code comprised programmatic assertions on every arithmetic path, an independent recomputation of the headline results from the raw response, and the complete re-execution described above. The detailed disclosure is available online [28], and the authors take full responsibility for the content.

Results

Study Selection and Characteristics

Screening excluded 31 of 524 records: three under E1 and 28 under E2, with no exclusions under E3 to E6, leaving 493 included studies (Figure 1).

**Figure 1:**
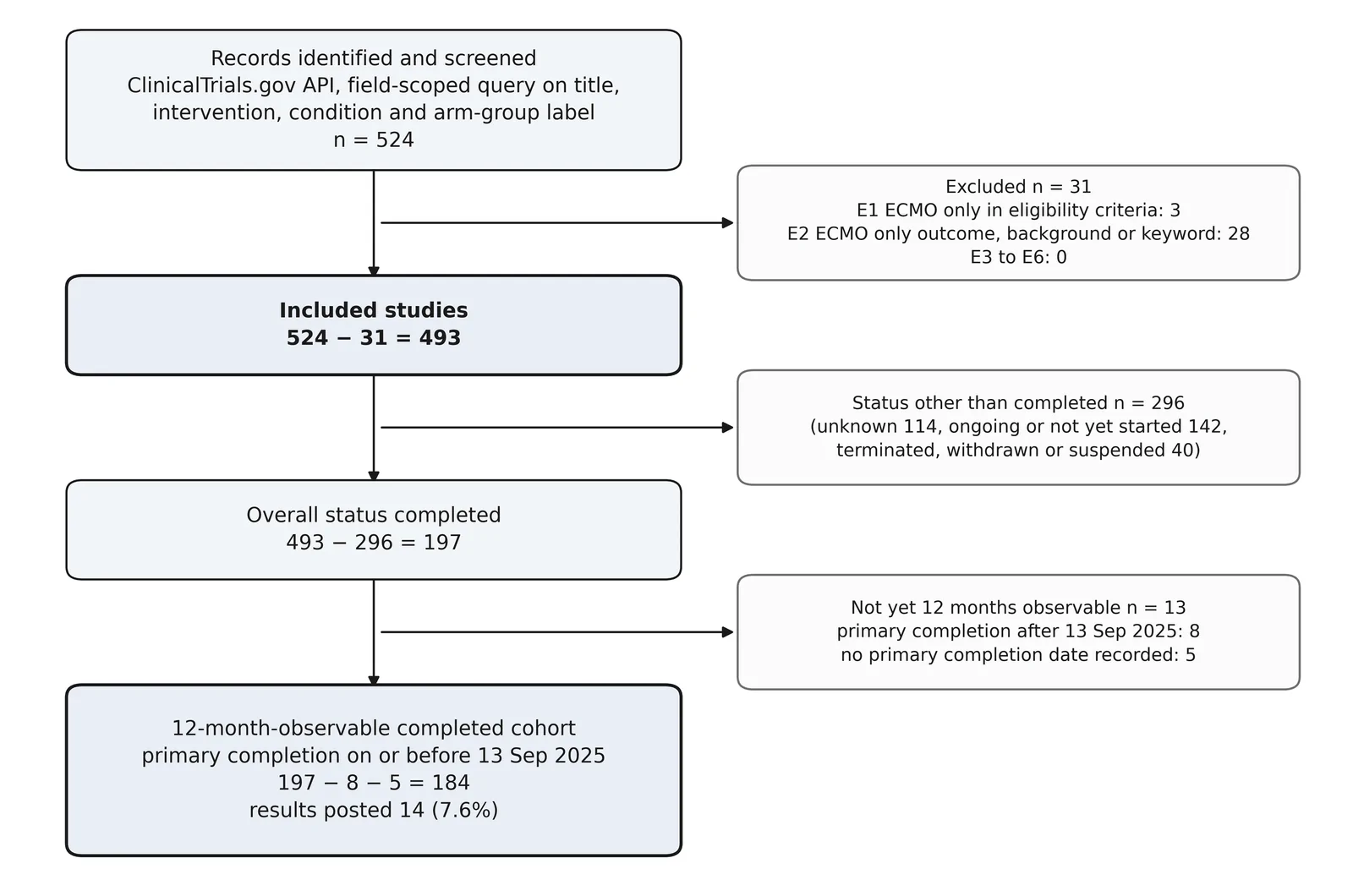
Study selection and derivation of the 12-month-observable completed cohort Records were retrieved from the ClinicalTrials.gov application programming interface on 13 September 2026 with a query restricted to title, intervention, condition and arm-group label fields. Exclusion codes are defined in the Methods; E3 (expanded access), E4 (duplicate registration), E5 (no human participants) and E6 (withdrawn without classifiable content) were each applied to no record. Status categories are given in full in Table 1. The broader all-field query used to check search sensitivity is described in the text.

Of these, 197 were completed; eight of those had a primary completion date after 13 September 2025 and five had none recorded, leaving the 12-month-observable completed cohort of 184. In the random sample of 100 broad-search records not returned by the field-scoped query, 82 named ECMO only in the eligibility or outcome text, and one met the eligibility criteria (1.0%, 95% CI 0.2 to 5.4): a recruiting paediatric pharmacokinetic study whose title misspells the term. With primary completion expected in 2029, it would not have entered the primary cohort. Extrapolated to the 979 unscreened records, the field-scoped search was estimated to have missed about 10 eligible studies (95% CI 2 to 53).

Table 1 describes study design, status and sponsorship.

**Table 1:** Design, status and lead sponsor of the 493 included studies Percentages are of the 493 included studies. Lead sponsor class is the registry's agency class; the other class includes one network and one individual record. The sponsor-name rows classify the 464 other-class records by the lead sponsor's recorded name; these names were not otherwise validated, and a non-industry lead sponsor does not exclude industry funding or collaboration. Allocation is not recorded for observational registrations. Census date: 13 September 2026.

Characteristic	Category	n	% of 493
Study type	Observational	268	54.4
Study type	Interventional	225	45.6
Allocation	Randomised	146	29.6
Allocation	Non-randomised	27	5.5
Allocation	Not applicable (single-group or sequential design)	52	10.5
Allocation	Not reported, observational registration	268	54.4
Overall status at census date	Completed	197	40
Overall status at census date	Unknown	114	23.1
Overall status at census date	Recruiting	92	18.7
Overall status at census date	Not yet recruiting	30	6.1
Overall status at census date	Terminated	20	4.1
Overall status at census date	Withdrawn	18	3.7
Overall status at census date	Active, not recruiting	16	3.2
Overall status at census date	Enrolling by invitation	4	0.8
Overall status at census date	Suspended	2	0.4
Lead sponsor class	Other class	464	94.1
Lead sponsor class	Industry	22	4.5
Lead sponsor class	Other government	5	1
Lead sponsor class	US National Institutes of Health	2	0.4
Other-class lead sponsor, by recorded name	University, hospital or research institute	406	82.4
Other-class lead sponsor, by recorded name	Other institution or network	22	4.5
Other-class lead sponsor, by recorded name	Named individual investigator	34	6.9
Other-class lead sponsor, by recorded name	Commercial company	2	0.4

A majority of the included studies were observational, 268 of 493 (54.4%), and 471 (95.5%) had a lead sponsor outside the industry class. Of the 464 records in the registry's other class, 406 named a university, hospital or research institute, 22 another institution or network, 34 a named individual investigator, and two a commercial company. For 114 studies (23.1%), status was unknown, meaning the record had passed its expected completion date without update.

Table 2 describes geography, sites and population.

**Table 2:** Geography, sites and population of the 493 included studies Percentages are of the 493 included studies. Region is assigned from listed location countries: records listing more than one country are multinational (23 records, 8 with a United States site) and records listing none are not reported. A site is a distinct listed facility. The enrolment rows give the median and quartiles of a continuous variable, for which a percentage is not applicable.

Characteristic	Category	n	% of 493
Region	Europe	217	44
Region	East Asia	103	20.9
Region	North America	79	16
Region	Other Asia / Middle East	16	3.2
Region	Oceania	12	2.4
Region	Latin America	2	0.4
Region	Africa	3	0.6
Region	Multinational	23	4.7
Region	Not reported	38	7.7
Sites	Single-site	366	74.2
Sites	Multi-site	89	18.1
Sites	Not reported	38	7.7
Age group enrolled	Adult only	396	80.3
Age group enrolled	Paediatric only	26	5.3
Age group enrolled	Both adults and children	71	14.4
Enrolment	Median, participants	55	not applicable
Enrolment	Lower quartile, participants	27.5	not applicable
Enrolment	Upper quartile, participants	152	not applicable
Enrolment	Records with an enrolment count	491	99.6

Studies were small and mostly single-site, with median enrolment 55 participants (interquartile range 27.5 to 152), and locations spanned 45 countries. Table 3 gives the clinical focus of the included studies.

**Table 3:** Clinical focus of the 493 included studies Condition category is assigned from the registry conditions field by a single prespecified precedence order (cardiac arrest, COVID-19, respiratory failure, cardiogenic shock, congenital heart disease, neonatal or paediatric, transplantation, trauma or poisoning, sepsis, coagulation, neurological injury, other), so categories are mutually exclusive and sum to 493. The full keyword dictionary is available online [28]. ECPR: Extracorporeal Cardiopulmonary Resuscitation; ARDS: Acute Respiratory Distress Syndrome. ​​

Condition category (precedence order)	n	% of 493
Cardiac arrest / ECPR	64	13
COVID-19	26	5.3
Respiratory failure / ARDS	113	22.9
Cardiogenic shock / heart failure	77	15.6
Congenital heart disease	0	0
Neonatal / paediatric-specific	11	2.2
Transplantation / organ preservation	10	2
Trauma / poisoning	4	0.8
Sepsis	15	3
Coagulation / haemostasis	27	5.5
Neurological injury	8	1.6
Other / mixed	138	28

Registrations rose steeply through the 2010s and peaked at 51 in 2021 (Figure 2).

**Figure 2:**
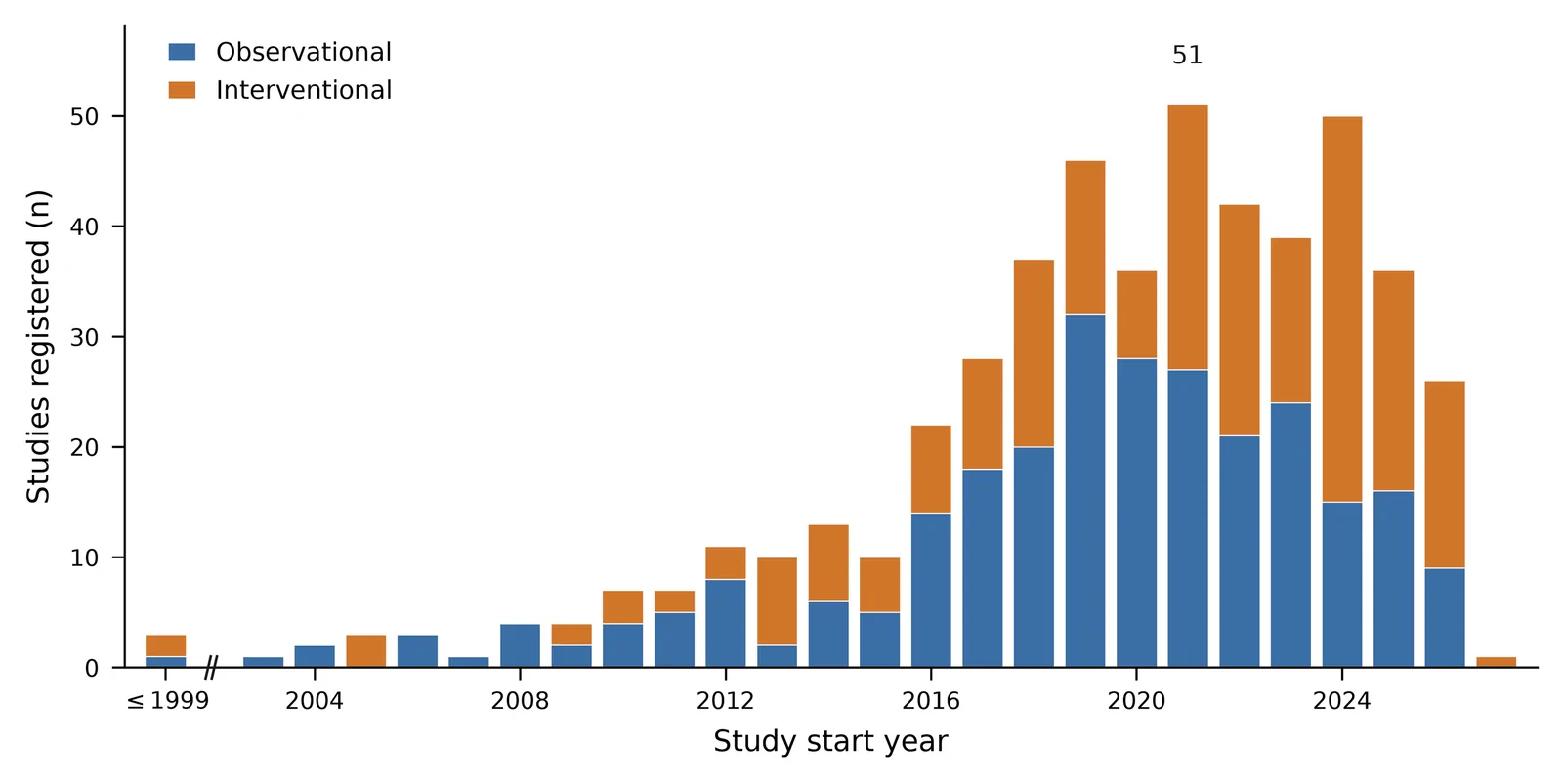
Registrations per study start year, by study type Stacked counts of the 493 included studies by recorded start year, observational in blue and interventional in orange. Studies starting before 2000 (3 studies, 1974, 1987 and 1996) are pooled in the leftmost bar. The 2026 and 2027 bars are incomplete years at the 13 September 2026 census date.

Primary Outcome

Of the 184 studies in the 12-month-observable completed cohort, 14 had posted summary results (7.6%, 95% CI 4.6 to 12.4). The cohort was 63.0% observational (116 of 184). No record among the 524 carried a delayed-submission certification. Table 4 reports the sensitivity analyses.

**Table 4:** Primary outcome and sensitivity analyses Each row recomputes the proportion of studies with posted summary results under one alternative definition (Wilson score 95% confidence intervals) [30]. Earliest and latest compatible dates replace each partial primary completion date with the first or last calendar day it could denote. The reversed-adjudication row resolves all 10 screening disagreements opposite to the checked decision. Terminated and unknown-status rows require a primary completion date on or before 13 September 2025.

Analysis	Posted (n)	Denominator (n)	Posted (%)	95% CI lower (%)	95% CI upper (%)
Primary analysis: completed, imputed partial dates	14	184	7.6	4.6	12.4
Earliest compatible primary completion date	14	184	7.6	4.6	12.4
Latest compatible primary completion date	14	184	7.6	4.6	12.4
Partially dated records excluded	10	150	6.7	3.7	11.8
All 10 screening adjudications reversed	14	183	7.7	4.6	12.4
Completed or terminated	19	203	9.4	6.1	14.2
Completed, terminated or unknown status	19	310	6.1	4	9.4
Terminated only	5	19	26.3	11.8	48.8
Unknown status only	0	107	0	0	3.5

Partial primary completion dates affected 34 records, four of them posters; no record crossed the eligibility boundary or the 12-month boundary under the earliest or latest compatible date, so the primary outcome was unchanged, and excluding partially dated records gave 10 of 150 (6.7%). Reversing all 10 screening adjudications gave 14 of 183 (7.7%). Including terminated studies gave 19 of 203 (9.4%), and further including studies of unknown status gave 19 of 310 (6.1%); none of the 107 unknown-status studies in the window had posted results.

Posting Across Study Characteristics

Table 5 and Figure 3 give posting by level of each characteristic, with risk differences, and Table 6 gives the single test for each characteristic.

**Table 5:** Results posting by level of each study characteristic, with risk differences Denominator is the 12-month-observable completed cohort of 184 studies; one study with no enrolment count is outside the tertile rows. Risk differences are percentage points against the stated reference level, with Newcombe hybrid score 95% confidence intervals [31]. All comparisons are exploratory, unadjusted and mutually confounded.

Characteristic	Level	Studies (n)	Posted (n)	Posted (%)	95% CI lower (%)	95% CI upper (%)	Reference level	Risk difference (percentage points)	Risk difference 95% CI lower	Risk difference 95% CI upper
Study type	Interventional	68	12	17.6	10.4	28.4	Observational	15.9	7.5	26.7
Study type	Observational	116	2	1.7	0.5	6.1	Observational	reference	reference	reference
Randomisation	Randomised	45	8	17.8	9.3	31.3	Not randomised	13.5	3.7	27.2
Randomisation	Not randomised	139	6	4.3	2	9.1	Not randomised	reference	reference	reference
Region	North America	30	9	30	16.7	47.9	Europe	25.9	11.3	44
Region	Europe	98	4	4.1	1.6	10	Europe	reference	reference	reference
Region	Other, multinational or not reported	31	1	3.2	0.6	16.2	Europe	-0.9	-7.4	12.3
Region	East Asia	25	0	0	0	13.3	Europe	-4.1	-10	9.5
Age group	Paediatric involved	45	6	13.3	6.3	26.2	Adult only or not reported	7.6	-1.2	20.7
Age group	Adult only or not reported	139	8	5.8	2.9	10.9	Adult only or not reported	reference	reference	reference
Enrolment tertile	T1 (36 or fewer)	67	12	17.9	10.6	28.7	T3 (more than 100)	17.9	7.8	28.7
Enrolment tertile	T2 (37 to 100)	65	2	3.1	0.8	10.5	T3 (more than 100)	3.1	-4.3	10.5
Enrolment tertile	T3 (more than 100)	51	0	0	0	7	T3 (more than 100)	reference	reference	reference

**Figure 3:**
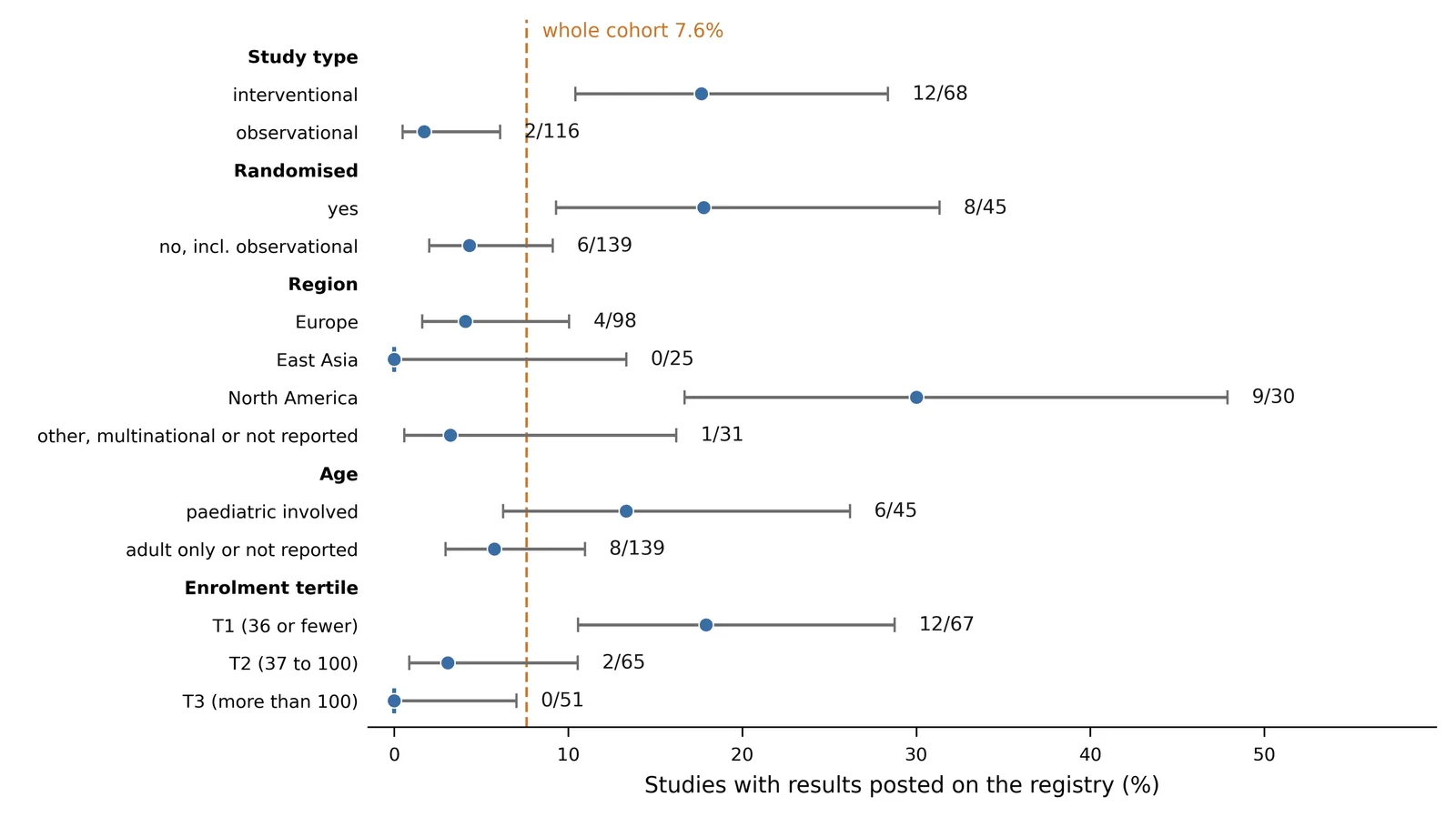
Results posting by study characteristic, with Wilson 95% confidence intervals Points are the proportions with posted summary results in the 12-month-observable completed cohort of 184 studies, and horizontal lines are Wilson score 95% confidence intervals [30]; a tick at zero marks a level with no posting. Levels are grouped by characteristic. Counts give studies posting over studies in the level. The dashed line is the whole-cohort proportion, 7.6%. Risk differences are in Table 5 and tests in Table 6.

**Table 6:** Hypothesis tests, one per study characteristic One test per characteristic compares the proportions posting across its levels in Table 5. For Fisher's exact and permutation tests, the chi-square statistic is descriptive of the contingency table only; the p value is the exact or permutation p value. No adjustment for multiplicity.

Characteristic	Test	Chi-square statistic (descriptive for exact tests)	Degrees of freedom	p value
Study type	Pearson chi-square	15.46	1	<0.001
Randomisation	Fisher exact, two-sided	8.76	1	0.007
Region	Monte Carlo permutation, 50,000 replicates	26.04	3	<0.001
Age group	Fisher exact, two-sided	2.78	1	0.11
Enrolment tertile	Monte Carlo permutation, 50,000 replicates	16.13	2	<0.001

Posting was 17.6% (12 of 68) in interventional and 1.7% (two of 116) in observational studies, a risk difference of 15.9 percentage points (95% CI 7.5 to 26.7), and 17.8% (eight of 45) in randomised studies against 4.3% (six of 139) in all others, 13.5 percentage points (3.7 to 27.2). Posting was 30.0% (nine of 30) among North American studies and 4.1% (four of 98) among European studies, and was lower in larger enrolment tertiles. These descriptive differences are confounded by study type, sponsor, intervention, calendar period and jurisdiction, and none can be attributed to regulation. In the Firth model with study type, North American location and log enrolment, the odds ratios were 6.93 (95% CI 1.84 to 37.83) for interventional design, 5.79 (1.54 to 23.96) for North American location and 0.62 per log unit of enrolment (0.32 to 1.14); the inverse association with enrolment seen in Table 5 was attenuated. In the model restricted to the prespecified reduced covariates, the odds ratio for enrolment was 0.39 per log unit (0.22 to 0.65) and for industry sponsorship 0.65 (0.005 to 6.56); the other-government class, with one study and no events, could not be estimated. Both models rest on 14 events and are exploratory.

Table 7 reports posting by lead sponsor class descriptively.

**Table 7:** Results posting by lead sponsor class (descriptive) Lead sponsor class is the agency class recorded by the registry. For each class, the table gives the proportion of studies in the 12-month-observable completed cohort that had posted summary results, with its Wilson score 95% confidence interval [30]. The National Institutes of Health class contributed no study to the 12-month-observable completed cohort, so no proportion was estimable and the cells record that.

Lead sponsor class	Included studies (n)	12-month-observable completed cohort (n)	Posted (n)	Posted (%)	95% CI lower (%)	95% CI upper (%)
Industry	22	8	0	0	0	32.4
US National Institutes of Health	2	0	0	not estimable, no study in cohort	not estimable	not estimable
Other government	5	1	0	0	0	79.3
Other class	464	175	14	8	4.8	13

Outside the other class every stratum had a single-digit denominator, and no comparative inference was drawn.

Timing of Results Submission and Posting

Among the 14 posters, the median interval from primary completion to first submission of results was 15.4 months (interquartile range 11.6 to 25.5), and to first posting 18.3 months (14.6 to 29.4); first posting followed first submission by a median of 86.5 days (range 24 to 169). Five studies submitted results within 12 months of primary completion, and one study posted within 12 months. Among the 170 non-posters, the median time from primary completion to the census date was 62.9 months (interquartile range 39.5 to 93.1; range 13.5 to 425.0), and 132 had passed 36 months. The Kaplan-Meier estimated proportion posting was 0.5% at 12 months and 7.6% at 36 months (Figure 4); the median time to posting was not reached.

**Figure 4:**
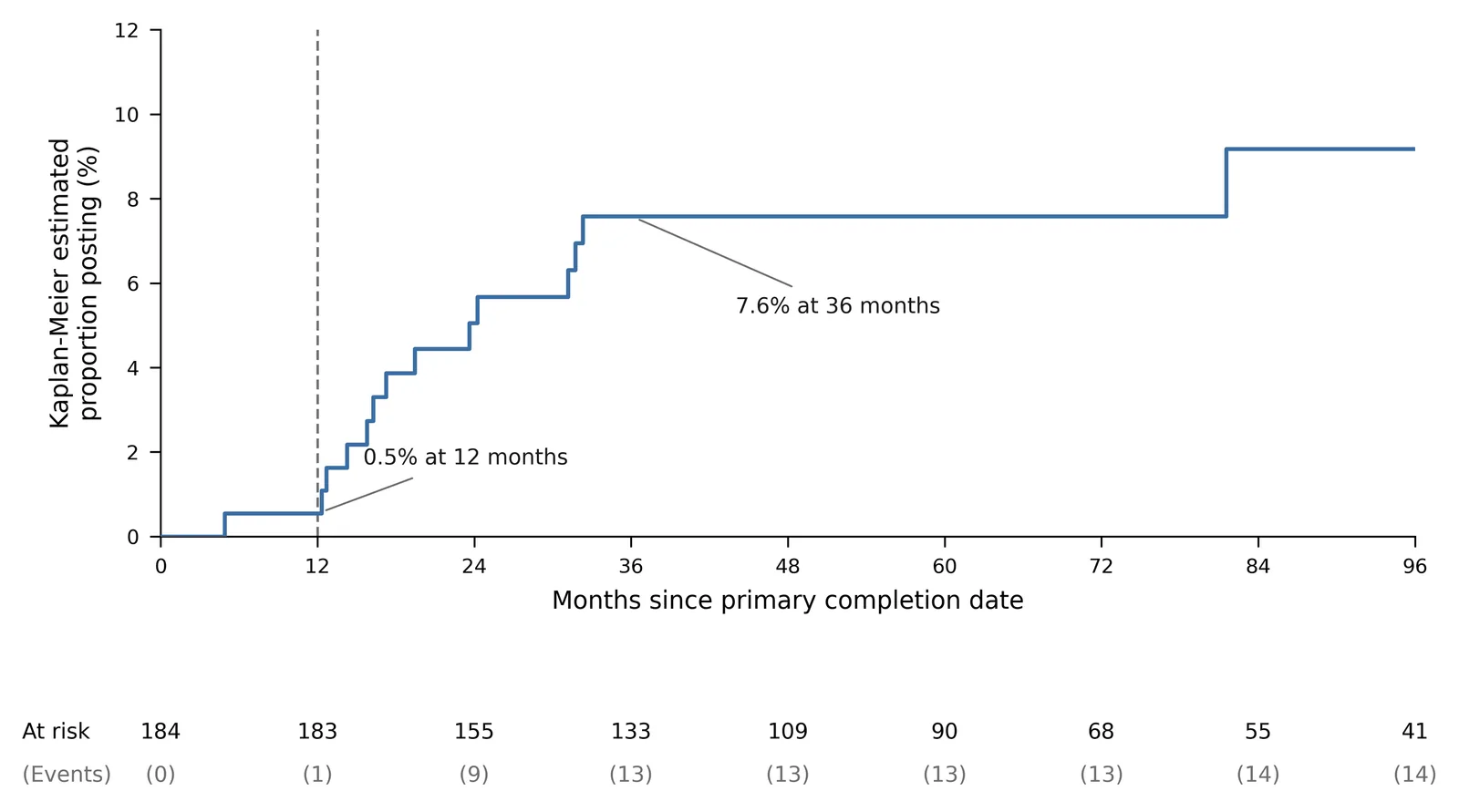
Kaplan-Meier estimated proportion posting results, by months since primary completion Kaplan-Meier product-limit estimate in the 12-month-observable completed cohort of 184 studies, with the recorded primary completion date as the time origin, results first posted as the event, and censoring at the 13 September 2026 census date; 14 events and 170 censored records. Numbers at risk and cumulative events are shown beneath the axis. The dashed line marks 12 months. The axis is truncated at 96 months; the last event occurred at 82 months.

Discontinuation

Forty of the 493 included studies (8.1%, 95% CI 6.0 to 10.9) were discontinued: 20 terminated, 18 withdrawn and two suspended (Table 8).

**Table 8:** Discontinued studies and coded reasons for stopping Percentages are of the 40 discontinued studies (terminated, withdrawn or suspended at the census date). Reasons follow the prespecified precedence order, so each study contributes to one category and the reason rows sum to 40. Examples of the verbatim text for each category and the full coding with both coders' assignments are deposited [28].

Category	Level	n	% of 40
Status at census date	Terminated	20	50
Status at census date	Withdrawn	18	45
Status at census date	Suspended	2	5
Coded reason for stopping	Slow accrual	12	30
Coded reason for stopping	Funding	7	17.5
Coded reason for stopping	Futility / interim	4	10
Coded reason for stopping	COVID-19	3	7.5
Coded reason for stopping	Investigator departure	3	7.5
Coded reason for stopping	Safety	1	2.5
Coded reason for stopping	Sponsor decision	0	0
Coded reason for stopping	Other	9	22.5
Coded reason for stopping	Not stated	1	2.5

Reasons were coded from the why-stopped text by prespecified patterns with every assignment read against the text; a second, model-assisted coding agreed on 35 of 40 records (kappa 0.85, 95% CI 0.72 to 0.97), and the five disagreements were resolved in the same model-assisted workflow without human review. Slow accrual was commonest (12 records). The nine records coded other comprised an unfavourable ethics decision, a protocol change with re-registration, loss of site collaborators or staff (2), a change in standard of care, an obsolete study, inability to achieve the primary objective (2) and a protocol never initiated.

Registry-Linked PubMed References

Sixty-one of the 184 studies (33.2%, 95% CI 26.8 to 40.2) carried at least one derived PubMed reference, 93 references in all (Table 9).

**Table 9:** Registry-linked PubMed references in the 12-month-observable completed cohort Derived references are PubMed records linked to the national clinical trial (NCT) number by the registry. Each was classified from its PubMed record; the classification of every reference is deposited [28]. Study-level rows count a study once, by the most informative article linked to it. These counts describe registry linkage; articles not indexed against the NCT number are not captured, so they are not a publication rate.

Level of count	Category	n	%
Reference level (93 references)	Primary results report of the registered study	58	62.4
Reference level (93 references)	Secondary, post hoc or follow-up analysis	21	22.6
Reference level (93 references)	Protocol or design paper	8	8.6
Reference level (93 references)	Not a report of the registered study	6	6.5
Study level (184 studies)	At least one derived PubMed reference	61	33.2
Study level (184 studies)	Linked to a primary results report	58	31.5
Study level (184 studies)	Linked only to secondary analyses	2	1.1
Study level (184 studies)	Linked only to a protocol	1	0.5
Study level (184 studies)	Posted results or linked results article	68	37
Study level (184 studies)	Both posted results and linked results article	6	3.3

Of these, 58 were primary results reports, 21 secondary, post hoc or follow-up analyses, eight protocol or design papers, and six were not reports of the registered study, including one linkage to an unrelated article. Sixty studies (32.6%, 95% CI 26.3 to 39.7) were linked to at least one article reporting their results, 58 to a primary results report, and one only to a protocol. Combining the two routes, 68 of 184 studies (37.0%, 95% CI 30.3 to 44.1) had either posted results or a linked results article; six had both.

Exploratory Post hoc Subgroup

Of the 68 interventional studies in the cohort, 52 completed on or after 18 January 2017, nine of those listed a United States site, and four met the intervention and phase criterion (Table 10).

**Table 10:** Exploratory post hoc subgroup approximating applicable clinical trial status Exploratory post hoc subgroup, not prespecified. All four criteria were required: interventional; primary completion on or after 18 January 2017; at least one United States location; and a drug or biologic intervention at phase 2, 3 or 4, or a device intervention. Registry fields cannot establish applicable clinical trial status [10]. Intervention type reproduces the registry field and may list more than one type.

National clinical trial (NCT) number	Lead sponsor	Intervention type	Phase	Enrolment	Primary completion	Results first posted
NCT03135210	University of Minnesota	DEVICE, OTHER	NA / not applicable	4	01-02-2020	21-02-2021
NCT03065647	University of Michigan	DEVICE	NA / not applicable	15	05-03-2020	15-03-2021
NCT03318393	University of Texas Southwestern Medical Center	DRUG	Phase 4	30	08-10-2021	31-01-2023
NCT05796557	Yale University	BIOLOGICAL	Phase 4	50	02-01-2025	31-05-2025

All four had posted results (Wilson 95% CI 51.0 to 100.0), against 10 of the other 180 (5.6%). The subgroup was too small, and its classification too uncertain, to support comparative or causal inference, and no formal comparison is reported.

Discussion

Among the 184 ClinicalTrials.gov-registered ECMO studies completed at least 12 months before the census date, 14 (7.6%) had posted summary results, and one had done so within 12 months of primary completion. A majority of the registered studies were observational and nearly all had a non-industry lead sponsor, consistent with the hypothesised composition of the field, although investigator initiation itself was not measured; many of these records are therefore unlikely to carry an FDAAA duty to post. Posting differed across recorded characteristics, being more common in interventional, randomised and North American studies, but these comparisons are descriptive and mutually confounded. About one third of the studies were linked to an article reporting their results.

These figures are low compared with audits of trials subject to the United States requirement [12-16], but the denominators differ. Those audits select trials to which the statute applies; the present cohort is an entire field in which most records are observational. The low posting proportion is therefore best read as describing how rarely the registry's results database is used in a field where most records are observational and probably outside the statutory scope, not as evidence of legal non-compliance. It is consistent with registry analyses of sepsis, stroke and heart failure research, where incomplete and delayed reporting was common [19-21].

Several explanations other than regulation could account for the variation observed. Interventional and randomised studies may have more resources, more experienced registry users, or funders and journals that expect deposition; North American studies may be more familiar with a United States registry; calendar period, study size and phase differ across strata. The four records meeting the post-hoc approximation all posted, but four approximately classified records cannot show that regulation produced reporting. In the penalised model, the inverse association between enrolment and posting was attenuated when study type and location were included, so it should not be read as an effect of size itself.

The publication indicator adds context. Classifying the linked references showed that most were genuine results reports, so where a link exists it usually leads to findings. But linkage depends on authors citing the NCT number in an indexed field, many publications do not, and no search beyond the registry's links was made. The 32.6% of studies linked to a results article is a floor and not a publication rate.

Nearly a quarter of included records had unknown status, and none of those in the window had posted results. Such records may represent completed work never reported, abandoned work never marked as stopped, or neglected records, and the registry does not distinguish these. Forty studies were discontinued, most often for slow accrual; this observation does not establish that single-centre design caused discontinuation.

Two practical points follow, and they are proposals rather than findings. First, journals and funders in critical care could ask for registry results deposition from observational as well as interventional studies, although ClinicalTrials.gov was designed principally around clinical trials, and discipline-specific repositories may suit some observational work. Second, ECMO research networks and registry infrastructure [1] could make deposition an expected output of participation.

Limitations

Studies never registered on ClinicalTrials.gov, or registered only elsewhere, are outside the sampling frame, so this study cannot estimate registration completeness. The field-scoped search missed an estimated 1% of the broad-search remainder. Screening passes were model-assisted, without calibration or a pilot, although every screening decision was checked by the first author. The post-review classification of linked PubMed references, the broad-search sample screen and the second coding of discontinuation reasons were model-assisted and not checked by a human reviewer, so they may contain misclassification. Registry fields are entered by sponsors and may be inaccurate or outdated, and sponsor class and region may be misclassified. Partial dates were imputed, although sensitivity analyses showed no effect on the primary outcome. Results submission can be distinguished from posting only for studies that posted, and delayed-submission certificates or extensions could not be verified beyond the registry's certification field. The applicable clinical trial approximation is unvalidated. The prespecified adjusted model was not computable and the penalised models rest on 14 events, so confounding, including by calendar time, is likely. Derived PubMed references are an incomplete measure of publication, and the linked proportion is a floor rather than a publication rate. The protocol was not registered, and its prospective specification cannot be independently verified. Finally, this is a single cross-section; the census date is stated so that the measurement can be repeated.

## Conclusions

Public posting of summary results on ClinicalTrials.gov was uncommon among completed ECMO studies. Most registered studies in this field were observational and had a non-industry lead sponsor, so many are probably outside the scope of the United States reporting requirement, and the low posting proportion should not be read as evidence of legal non-compliance. Posting was more frequent among interventional, randomised and North American studies, but these comparisons were exploratory and mutually confounded. The small group of records that approximated applicable clinical trial status had all posted results, yet this subgroup was too small, and its classification too uncertain, to support conclusions about regulatory effects. Only a minority of studies were linked through the registry to an article reporting their results, and this linkage is an incomplete measure of publication. A substantial share of records had an unknown status, which leaves the fate of those studies unclear. Further work using verified legal status and comprehensive publication matching is needed to establish how completely the findings of this field reach the public.
